# Adverse event reporting in randomised trials of neuropathic pain: challenges for clinical usefulness of safety data

**DOI:** 10.1186/1745-6215-12-S1-A12

**Published:** 2011-12-13

**Authors:** Victoria R Cornelius, Odile Sauzet, Salma Ayis, Joy Ross, Paul Farquhar-Smith, Ruth A Branford, John E Williams, Janet L Peacock

**Affiliations:** 1 King’s College London, London, SE1 3QD, UK; 2 University of Southampton, Southampton, SO16 6YD, UK; 3 Royal Marsden Hospital, London, SW3 6JJ, UK; 4 St Joseph’s Hospice Hackney, London, E8 4SA, UK

## Background

Monitoring the safety of therapies is of paramount importance in protecting patients from harm and enabling risk-benefit assessment. The recording and reporting of measures of efficacy has received considerable attention and while by no means perfect, has advanced further than the parallel assessment of harm. The stimulus for this study came from a commissioned effectiveness and cost-effectiveness review of treatments for neuropathic pain in patients (the CEAN study) [1]. CEAN noted that the completeness of adverse event (AE) reporting varied between trials and some expert opinion was required where primary data were insufficient for modeling cost-effectiveness. Further, clinicians indicated that trials sometimes failed to provide adequate information for clinical decision-making and informing patients.

## Objectives

To describe how AE data are collected and reported. To explore results post-2004 (when regulatory requirements regarding collection and reporting of AEs for RCTs were in place)

## Methods

Relevant CEAN study publications (RCTs of anticonvulsants and antidepressants for post herpetic neuralgia and painful diabetic neuropathy) with separable primary data on impact on pain. Items for data extraction were generated using recommendations set out in CONSORT 2004 [2]. Additional information extracted sought to determine the criteria used by authors to select AEs for reporting (e.g. significant differences), the mode of collection (e.g. observation or questionnaire) and how they were collected (e.g. passively, actively (prompted). Double data extraction was performed.

## Results

53 publications were included, 12 were published post 2004. Key results are presented in Tables 1 and 2. A subset of the recommendations laid out in CONSORT 2004 were not adhered to by any of the publications. The collection method impacts on the number of AEs reported by patients and this was poorly reported by the majority of trials. The criteria used by authors for reporting AEs varied substantially across publications.

**Table 1:** 

	All (N=53) n (%)	Post-2004 (N=12) n (%)
Reported total number who withdrew & withdrew due to AE	48 (91)	12 (100%)
Reported that grading for AEs were assigned	30 (57)	9 (75)
Reported mode of collection (e.g. questionnaire, patient reported, observation)	23 (43)	5 (42)
Distinction between severe/life threatening AEs and those that were not.	29 (55)	11 (92)
Reported the dictionary used for coding AEs	1 (1.9)	0 (0.0)

**Table 2:** Criteria used to select AEs for reporting

Criteria	n (%)
Most frequent AEs	17 (32)
All AE’s that occurred	17 (32)
Other	6 (11)
A pre-specified list of AEs	5 (9.4)
Unclear	3 (5.7)
Not applicable as no AE reported	3 (5.7)
Any AE with sig diff between treatment groups	2 (3.8)

## Conclusion

Synthesis of AE data across studies is hampered by the lack of information on collection methods and by arbitrary heterogeneous criteria used by authors for selecting AEs to be reported. In order to improve the usefulness of AE data reported by publications, validated methods for collection need to be developed, and core AEs for reporting need to be agreed. Online journal supplements can be utilised to overcome journal space limitations. The issues highlighted by this case study are likely to be relevant for AE reporting in RCTs in general, although solutions will likely need to be tailored to specific therapeutic-disease areas.
